# Immune landscape and prognostic immune-related genes in *KRAS*-mutant colorectal cancer patients

**DOI:** 10.1186/s12967-020-02638-9

**Published:** 2021-01-07

**Authors:** Jungang Liu, Xiaoliang Huang, Haizhou Liu, Chunyin Wei, Haiming Ru, Haiquan Qin, Hao Lai, Yongsheng Meng, Guo Wu, Weishun Xie, Xianwei Mo, Caroline H. Johnson, Yawei Zhang, Weizhong Tang

**Affiliations:** 1 Division of Colorectal & Anal Surgery, Department of Gastrointestinal Surgery, Guangxi Medical University Cancer Hospital, Nanning, 530021 Guangxi Zhuang Autonomous Region People’s Republic of China; 2 Guangxi Clinical Research Center for Colorectal Cancer, Nanning, Guangxi Zhuang Autonomous Region People’s Republic of China; 3 Department of Research, Guangxi Medical University Cancer Hospital, Nanning, Guangxi Zhuang Autonomous Region People’s Republic of China; 4 Department of Environmental Health Sciences, Yale School of Public Health, 60 College Street, New Haven, CT 06520 USA

**Keywords:** Colorectal cancer, Tumor-infiltrating immune cells, Immunosuppression, *KRAS* mutation

## Abstract

**Background:**

*KRAS* gene is the most common type of mutation reported in colorectal cancer (CRC). *KRAS* mutation-mediated regulation of immunophenotype and immune pathways in CRC remains to be elucidated.

**Methods:**

535 CRC patients were used to compare the expression of immune-related genes (IRGs) and the abundance of tumor-infiltrating immune cells (TIICs) in the tumor microenvironment between *KRAS*-mutant and *KRAS* wild-type CRC patients. An independent dataset included 566 cases of CRC and an in-house RNA sequencing dataset were served as validation sets. An in-house dataset consisting of 335 CRC patients were used to analyze systemic immune and inflammatory state in the presence of *KRAS* mutation. An immue risk (Imm-R) model consist of IRG and TIICs for prognostic prediction in *KRAS*-mutant CRC patients was established and validated.

**Results:**

NF-κB and T-cell receptor signaling pathways were significantly inhibited in *KRAS*-mutant CRC patients. Regulatory T cells (Tregs) was increased while macrophage M1 and activated CD4 memory T cell was decreased in *KRAS*-mutant CRC. Prognosis correlated with enhanced Tregs, macrophage M1 and activated CD4 memory T cell and was validated. Serum levels of hypersensitive C-reactive protein (hs-CRP), CRP, and IgM were significantly decreased in *KRAS*-mutant compared to *KRAS* wild-type CRC patients. An immune risk model composed of VGF, RLN3, CT45A1 and TIICs signature classified CRC patients with distinct clinical outcomes.

**Conclusions:**

*KRAS* mutation in CRC was associated with suppressed immune pathways and immune infiltration. The aberrant immune pathways and immune cells help to understand the tumor immune microenvironments in *KRAS*-mutant CRC patients.

## Background

Colorectal cancer (CRC) remains a major cause of cancer-related mortality worldwide despite advancements in tumor screening, early diagnosis, and curative resection. Currently, radical resection is the sole reliable method of cure for CRC. At the diagnostic stage, 20–25% of CRC patients show evidence of metastatic disease with no scope for radical surgery [1]. Subsequent to curative resection, the recurrence rate of metastasis in patients is approximately 70%, of which 50% are fatal [2]. Survival in patients with untreated metastatic CRC is around six months. Treatment regimens combining cytotoxic chemotherapy and biological agents improved overall survival of patients with metastatic CRC by more than two years [3]. The advent of immunotherapy further advanced the scope of prolonging survival in cancer patients. Immune checkpoint blockade therapy has shown promising therapeutic results in patients with advanced malignant tumor, such as non-small cell lung cancer, melanoma, renal cell carcinoma, and mismatch repair-deficient tumors [4, 5]. Immune checkpoint blockade therapy has been beneficial in microsatellite instability-high CRC patients [6], which account for 15% of all CRCs [7]. However, since the majority of CRC patients are microsatellite-stable, researching the immune microenvironment and identifying potential immunotherapeutic targets are important in improving the effectiveness of immunotherapy in these patients.

In CRC, the common canonical gain-of-function mutation is the oncogenic mutation of Kirsten rat sarcoma viral oncogene homolog (*KRAS*) that encodes GTPases, namely, KRAS4A and KRAS4B [8]. RAS, a key molecule of the mitogen-activated protein kinase (MAPK) signaling pathway, is activated by the binding of ligands such as the vascular endothelial growth factor (VEGF) to receptor tyrosine kinases (RTKs). RAS exists in two states, the active (GTP, guanosine triphosphate) or non-active-forms (GDP, guanosine diphosphate). Transition between the two states is responsible for the signal transduction crucial for cell growth and differentiation [9]. *RAS* mutations lead to persistent activation of multiple downstream effectors resulting in the induction of malignant transformation [10]. The prevalence of *KRAS* mutations in CRC patients is approximately 30–50% and is associated with poor prognosis and metastasis [11]. Clinical significance of *KRAS* mutation is proved by its use as a biomarker of EGFR-TKI resistance and its application in identifying suitable patients for anti-epidermal growth factor receptor (EGFR) therapies [12].

Current research evidence suggests a significant influence of *KRAS* mutation in tumor immunity. An unsupervised hierarchical clustering analysis of immune genes/signatures named the Co-ordinate Immune Response Cluster (CIRC), comprising 28 genes, revealed a relatively high proportion of patients with *KRAS* mutation in the cluster associated with low inhibitory molecule expression [13, 14]. In addition, immunophenotyping of colon tumors from mice indicated an association between *Kras* mutation and an immunosuppressive microenvironment characterized by decreased T-cell infiltration and increased infiltration of myeloid-derived suppressor cells (MDSCs) [15]. However, the immune landscape and altered expression of immune-related genes in CRC patients with *KRAS* mutation have not been fully elucidated.

The present study systematically depicts the immune landscape, profiles immune-related genes (IRGs), and compares systemic immune markers between *KRAS*-mutant and *KRAS* wild-type CRC patients based on TCGA, GEO and in-house dataset. Our results indicate an association of *KRAS* mutation with local and systemic immunosuppression in CRC. In addition, an immue risk (Imm-R) model was established, which was associated with immune infiltration and prognosis in CRC patients.

## Materials and methods

### Clinical specimens

In the present study, 8 cases of CRC samples including 3 cased of *KRAS*-mutant CRC samples and 5 cased of *KRAS* wild-type CRC samples were obtained from patients at the Guangxi Medical University Cancer Hospital. The samples were subjected to RNA sequencing. All of the patients were pathologically diagnosed as CRC without chemotherapy or radiotherapy before the collection of the tissues. Written informed consents were obtained from all patients. The study was approved by the Ethics and Human Subject Committee of Guangxi Medical University Cancer Hospital. All experiments and methods were performed according to relevant guidelines and regulations formulated by the Guangxi Medical University.

### RNA-seq analysis

Total RNA was extracted using Trizol reagent (Invitrogen). The construction of RNA-seq library was based on the protocol of the IlluminaTruSeq RNA Sample Preparation Kit (illumina). Finally, RNA-seq analysis was performed by GENE + company (Beijing, China) using Illumina HiSeqX Ten platforms. After quality control and trimming adaptor, reads were mapped onto human genome GRCh38. RNA-seq data have been deposited in the China National Center for Bioinformation (ID: PRJCA003751).

### Data acquisition and processing

High-throughput RNA sequencing and somatic mutation data (VarScan2 Variant Aggregation and Masking) related to colon and rectal adenocarcinoma available in The Cancer Genome Atlas (TCGA) were downloaded from the GDC Data Portal (https://portal.gdc.cancer.gov/) on July 25, 2019. Totally, 535 patients with complete somatic mutation data were included in this study. Of 535 CRC patients, 528 had high-throughput RNA sequencing data. Gene symbols were annotated based on GRCh38.91. Mutation Annotation Format (MAF) files of colon and rectal adenocarcinoma were merged together to obtain somatic mutation data. Visualization and summarization of somatic mutation data was achieved using the Maftools R package [16].The corresponding clinical information of the patients was simultaneously downloaded. The GSE39582 dataset from the Gene Expression Omnibus (GEO, https://www.ncbi.nlm.nih.gov/geo/) repository was downloaded, which included gene expression profile of 566 cases, and served as an independent validation dataset. Gene IDs were transformed using the clusterProfiler R package [17].

### In-house data collection

Clinical and pathological data from CRC patients hospitalized at the Guangxi Medical University Cancer Hospital (Nanning, China) between July 2013 and October 2018 were documented. The criteria for inclusion of patients in this study were as follows: (i) pathologically confirmed CRC and (ii) primary tumor resection with *KRAS* mutation detected based on postoperative gross specimen analysis. The exclusion criteria were: (i) exposure to prior preoperative therapy (including radiotherapy, chemotherapy, or chemoradiotherapy), (ii) with other types of cancer before or after CRC diagnosis, (iii) with known familial adenomatous polyposis or hereditary non-polyposis colorectal cancer, and (iv) diagnosed with infectious diseases or systemic stress reaction at the time of first admission to hospital.

Medical records of patients were examined to document information related to demographic and clinical characteristics such as age, sex, pathological stage, preoperative routine blood test, serum Igs, complement proteins C3 and C4, C-reactive protein (CRP), high-sensitivity C-reactive protein (hs-CRP), percentage of T lymphocytes, B lymphocytes, and natural killer (NK) cells, and *KRAS* mutation status. Based on the inclusion and exclusion criteria, 335 CRC patients (101 patients with *KRAS* mutation and 234 patients with *KRAS* wild-type tumors) were enrolled in the current study. The protocol of this retrospective study was approved by the Ethics and Human Subject Committee of Guangxi Medical University Cancer Hospital, and all experiments and methods met the standards of the relevant guidelines and regulations.

### Generation of IRGs list

The list of IRGs was collected from the immunology database and analysis portal (ImmPort) and TISIDB [18, 19]. The ImmPort database encompasses accurately updated information related to immunology and provides a list of IRGs curated with functions and Gene Ontology terms. TISIDB is a web portal that facilitates comprehensive investigation of tumor-immune interactions and provides a list of genes associated with anti-tumor immunity reported in literature. IRGs enlisted in ImmPort and TISIDB are from different sources that complement each other. In the present study, IRGs listed in both the databases were amalgamated and the genes that were annotated by the Ensembl database were retained. Accordingly, the IRGs list in the current study was made up of 1951 genes.

### Estimation of the abundance of immune cells

The tumor immune estimation resource (TIMER) is a web-accessible resource that estimates the abundance of six types of tumor-infiltrating immune cells (TIICs) (B cells, CD4 and CD8 T cells, neutrophils, macrophages, and dendritic cells) [20]. TIMER deduces the abundance of TIICs from gene expression profiles based on a deconvolution method validated by Monte Carlo simulations, orthogonal estimates from DNA methylation-based inferences, and pathological assessments. CIBERSORT is a deconvolution algorithm based on support vector regression, which uses a set of reference gene-expression values corresponding to a minimal representation for each cell type to infer cell type proportions in data from bulk tumor samples with mixed cell types [21]. CIBERSORT could sensitively and specifically discriminate 22 human immune cell phenotypes. The abundance of TIICs was analyzed using TIMER in 528 CRC patients (Seven patients without high-throughput RNA sequencing data were excluded from 535 CRC patients) enrolled in the study and then validated by CIBERSORT using the default parameters.

### Construction and validation of tumor-infiltrating immune cell(TIICs)signature

The TCGA dataset served as the training set, and GSE39582 was the validation set. In the training set, we first applied the univariable survival analysis to define the prognostic value of the TIICs in patients with *KRAS* mutation. TIICs with significant prognostic value were first validated in the validation set. TIICs with significant prognostic value in both training set and validation set were analyzed using the multivariate Cox proportional hazards regression model, and those with a *p* value < 0.05 were used to construct the TIICs signature. A formula for the TIICs signature was established to predict patient survival: TIICs signature = ∑Cox coefficient of TIIC Xi × abundance of TIIC Xi. The prognostic performance of the TIICs signature was evaluated using the receiver operating characteristic (ROC) curve and area under the curve (AUC).

### Identification of differentially expressed IRGs

To identify IRGs associated with *KRAS* mutation in CRC, the expression of 1951 IRGs between *KRAS*-mutant and *KRAS* wild-type CRC was compared using the R software package empirical analysis of digital gene expression data in R (edgeR) [22]. Trimmed mean of M-values (TMM) method was used to normalize the count data in edgeR. The threshold for filtering differentially expressed IRGs was set at a false discovery rate (FDR) of < 0.05 and a log_2_ fold change > 1.

### Functional enrichment analysis

The pathways and biological processes affected by *KRAS* mutation in CRC were identified by gene set enrichment analysis (GSEA) using the clusterProfiler R package. A list of sorted genes obtained based on the fold-change of mean expression of the genes between *KRAS*-mutant and *KRAS* wild-type CRC patients represented the input file. Biological processes were evaluated using the Kyoto Encyclopedia of Genes and Genomes (KEGG) pathways and gene ontology (GO).

### Construction of an immune risk (Imm-R) model

The expression profile of differentially expressed IRGs between *KRAS*-mutant and *KRAS* wild-type CRC was analyzed using univariate Cox regression analysis. Input dataset was in log_2_ (normalized value + 1) data format. The prognostic value of differentially expressed IRGs for overall survival (OS) was defined by univariate Cox regression analysis wherein genes were regarded as significant at *p* < 0.05. IRGs identified as prognostic indicators by univariate Cox regression analysis were subsequently subjected to multiple Cox regression analysis. IRGs identified as independent prognostic indicators in multiple Cox regression analysis together with TIICs signature were used to construct the Imm-R model. The individual risk value was calculated by multiplying the expression value of each prognostic indicator and the cox regression coefficient. The association between the risk value and immune infiltration was analyzed by comparing the abundance of immune cells between the high- and low-risk groups.

### Statistical analysis

All statistical analyses were performed using the R software (Version: 3.5.0). OS between two groups was compared using the Kaplan–Meier survival analysis and log-rank test. Spearman rank correlation test was used to evaluate the correlation between the expression of IRGs and abundance of immune cells. Results with two-sided *p* < 0.05 were considered statistically significant.

## Results

### Somatic mutation landscape of CRC patients

**Fig. 1:**
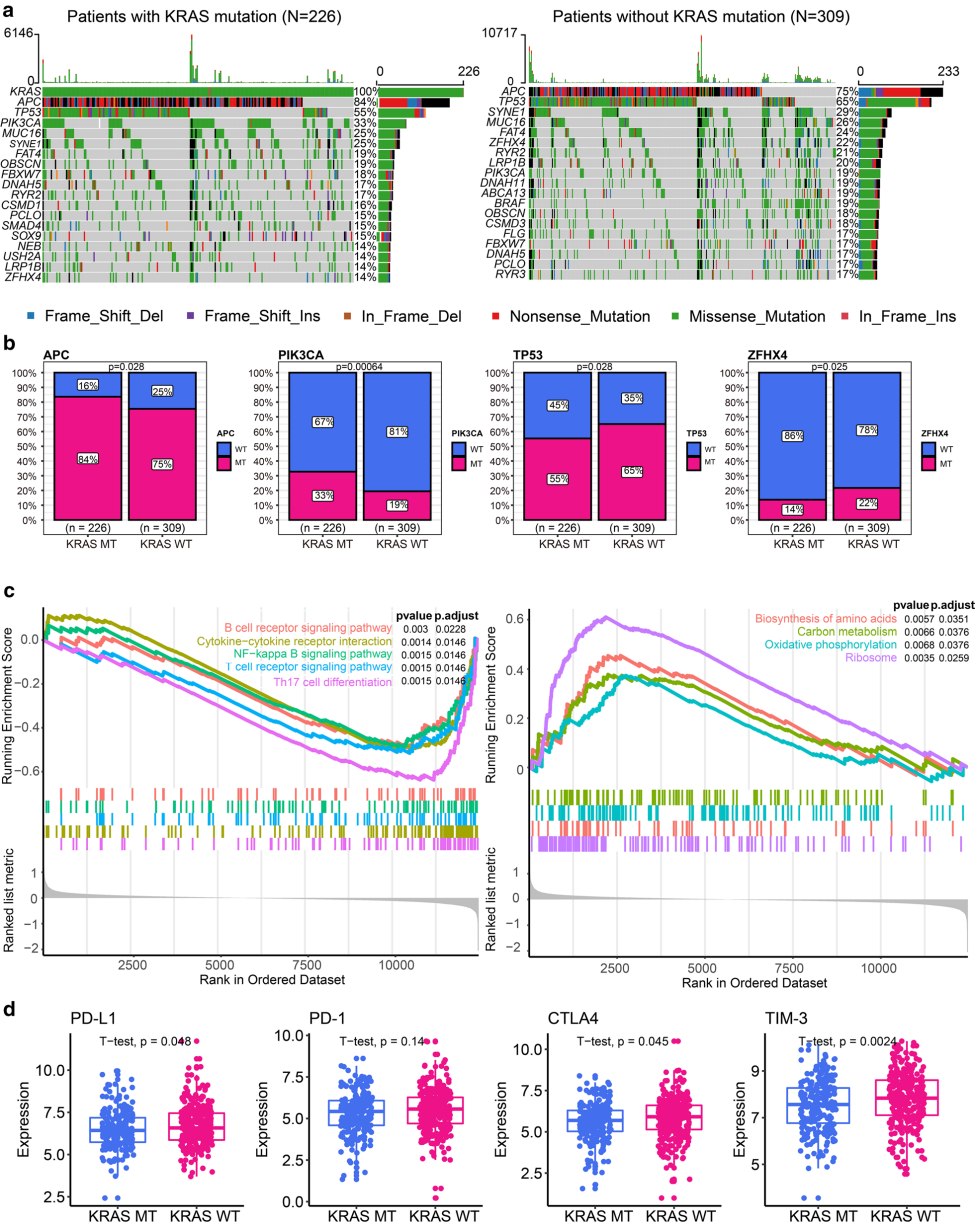
Somatic mutation landscape of colorectal cancer (CRC) patients based on KRAS status.** a** Somatic mutation landscape of CRC patients with (left panel) or without (right panel) *KRAS* mutation. Seven common mutation types were counted. **b** Genes with significantly different mutation frequencies between the *KRAS* mutant and *KRAS* wild-type group. **c** Significantly different pathways between *KRAS* mutant and *KRAS* wild-type groups. Pathways significantly down regulated in *KRAS*-mutant CRC patients (left) and significantly up regulated in *KRAS*-mutant CRC patients (right). **d** The expression of key immune checkpoint-associated molecules between *KRAS* mutant and *KRAS* wild-type groups

Somatic mutation landscape analysis was performed to analyze the status of *KRAS* in 535 CRC patients. Of them, 99.63% were detected to possess at least one type of gene mutation (Additional file 1: Figure S1). The most frequent mutation seen in CRC was in the adenomatous polyposis coli (*APC*) gene (79%) followed by the tumor protein 53 (*TP53*) gene (61%). *KRAS* mutation was the third common mutation detected in CRC with a frequency of 42%. We divided patients into two groups based on the presence or absence of *KRAS* mutations. The genes with the top 3 mutation frequencies (except for *KRAS*) in the *KRAS* mutant group were APC, TP53 and PIK3CA. The genes with the top 3 mutation frequencies in *KRAS* wild-type patients were APC, TP53 and SYNE1 (Fig. 1a). We compared the frequency of gene mutations between the two groups and found that mutation frequency of APC and PIK3CA was significantly increased in the *KRAS* mutant group, while mutation frequency of TP53 and ZFHX4 was significantly increased in the *KRAS* wild-type group (Fig. 1b). *KRAS* mutation-induced altered pathways were evaluated using GSEA based on the data obtained from *KRAS*-mutant and *KRAS* wild-type CRC patients. Several immune-related pathways were significantly down-regulated in *KRAS*-mutant compared to that of *KRAS* wild-type CRC patients, namely, Th1 and Th2 cell differentiation, T cell receptor signaling, and nuclear factor kappa-B (NF-κB) signaling pathways (Fig. 1c). Four pathways were significantly up regulated in *KRAS*-mutant compared to that of *KRAS* wild-type CRC patients, namely, biosynthesis of amino acids, carbon metabolism, oxidative phosphorylation and ribosome (Fig. 1c). GSEA based on biological processes and molecular function revealed inhibition of several immune-related terms in *KRAS*-mutant compared to that of *KRAS* wild-type CRC patients (Additional file 2: Figure S2). Taken together, these data indicate an association of *KRAS* mutation with immune-suppression in CRC. The expression of immune checkpoint molecules, such as PD-1, are a promising predictive factor for immune treatment response. We explored the association between expression of immune checkpoint molecules and *KRAS* mutation. Interestingly, we found that several key immune checkpoint-associated molecules (PD-L1, CTLA4 and TIM-3, all *p* < 0.05, Fig. 1d) were significantly downregulated in the *KRAS* mutant group. We speculated *KRAS* mutations might inhibit checkpoint molecules.

### Immune landscape of CRC patients in the presence and absence of *KRAS* mutation

**Fig. 2:**
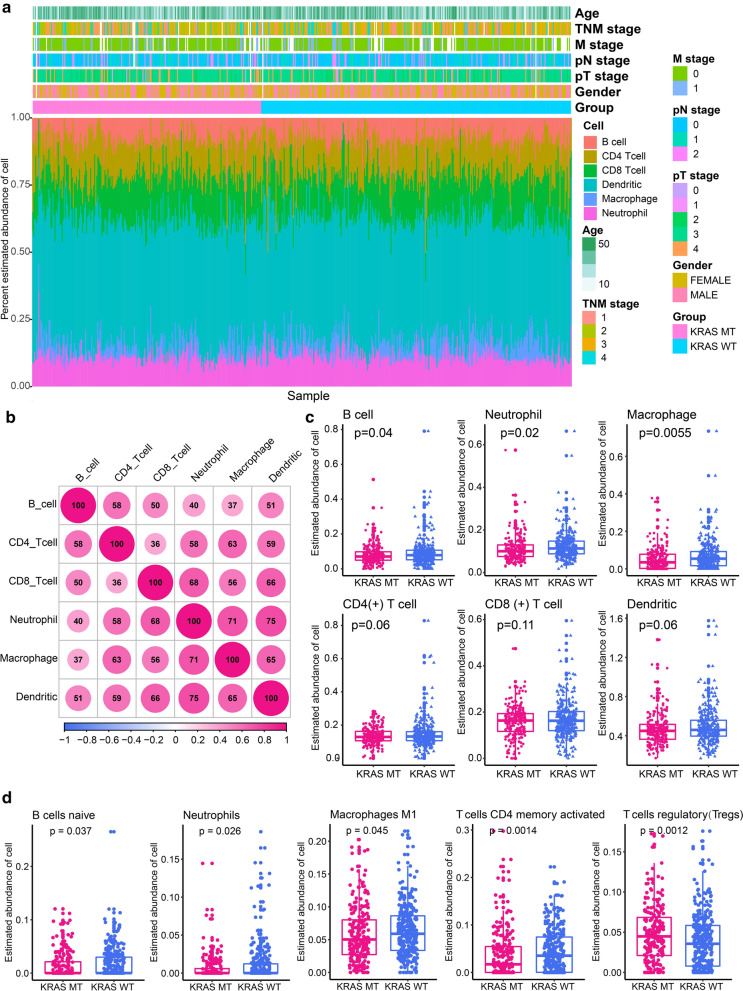
Immune landscape of colorectal cancer (CRC) patients in the presence and absence of *KRAS* mutation.** a** Percentage abundance of six types of tumor-infiltrating immune cells. **b** Correlation matrix showing abundance of six types of immune cells. Correlation coefficients displayed are expanded 100 times. **c** Abundance of six types of tumor-infiltrating immune cells in *KRAS*-mutant and *KRAS* wild-type CRC patients. **d** Differential abundance of tumor-infiltrating immune cells between the *KRAS*-mutant and *KRAS* wild-type CRC

The immune landscape was successfully analyzed among 528 CRC patients (*KRAS* mutant: 224; *KRAS* wild-type: 304). We comprehensively compared the spectrum of immune cell infiltration in the presence and absence of *KRAS* mutation. As shown in Fig. 2a, significant variations were observed in the proportion of TIICs among different individuals. Thus, variation in the proportion of TIICs represent intrinsic characteristics that underlie individual differences. Correlation analysis revealed different subpopulations of immune cells displaying weak to moderate (correlation coefficient ranging from 0.36 to 0.75) positive correlation (Fig. 2b). The abundance of different types of immune cells was compared between the *KRAS*-mutant and *KRAS*-wild type CRC patients (Fig. 2c). The abundance of B cells (0.08 ± 0.06 vs. 0.09 ± 0.08), neutrophils (0.11 ± 0.06 vs. 0.13 ± 0.07), and macrophage (0.05 ± 0.06 vs. 0.07 ± 0.08) were significantly down-regulated in *KRAS*-mutant compared to that of *KRAS* wild-type CRC patients. Considering that immune cells have multiple subtypes, we used CIBERSORT for further typing of immune cells and validation the results of TIMER. The abundance of 22 immune cells estimated by CIBERSORT was shown in Additional file 3: Figure S3. Comparing the abundance of 22 immune cells between the *KRAS*-mutant and *KRAS* wild-type groups, we found that native B cells, neutrophils and macrophage M1 were significantly down-regulated in the *KRAS*-mutant group (all *p* < 0.05, Fig. 2d), which was a further refinement of the results of TIMER. In addition, we observed that activated CD4 memory T cell was significantly decreased but regulatory T cells (Tregs) was significantly increased in the *KRAS*-mutant group (Fig. 2d). Microsatellite instability-high (MSI-H) or mismatch repair deficient (dMMR) CRC exhibit an active immune microenvironment due to the hyper-mutated state of the tumor cells [23]. We excluded samples with MSI-H or dMMR and re-analyzed the differences in the immune microenvironment between the two groups. Totally, 72 cases (13.63%) of CRC with MSI-H or dMMR were excluded, including 21 *KRAS* mutant and 51 *KRAS* wild-type. We found that four of the above five TIICs (native B cells, macrophage M1, activated CD4 memory T cell and Tregs), except neutrophils, had significant differences between the two groups (Additional file 4: Figure S4a). We further used in-house RNA sequencing data to compare the abundance of the above five TIICs in the *KRAS*-mutant and *KRAS* wild-type groups (*KRAS*-mutant groups: n = 3; *KRAS* wild-type groups: n = 5). Macrophage M1 was also significantly down-regulated in the *KRAS*-mutant group (*p* = 0.039, Additional file 4: Figure S4b). For several other TIICs, we observed similar trends to the above results, but the differences were not statistically significant due to the small sample size.

### Development and validation of the tumor-infiltrating immune cell(TIICs)signature

**Fig. 3:**
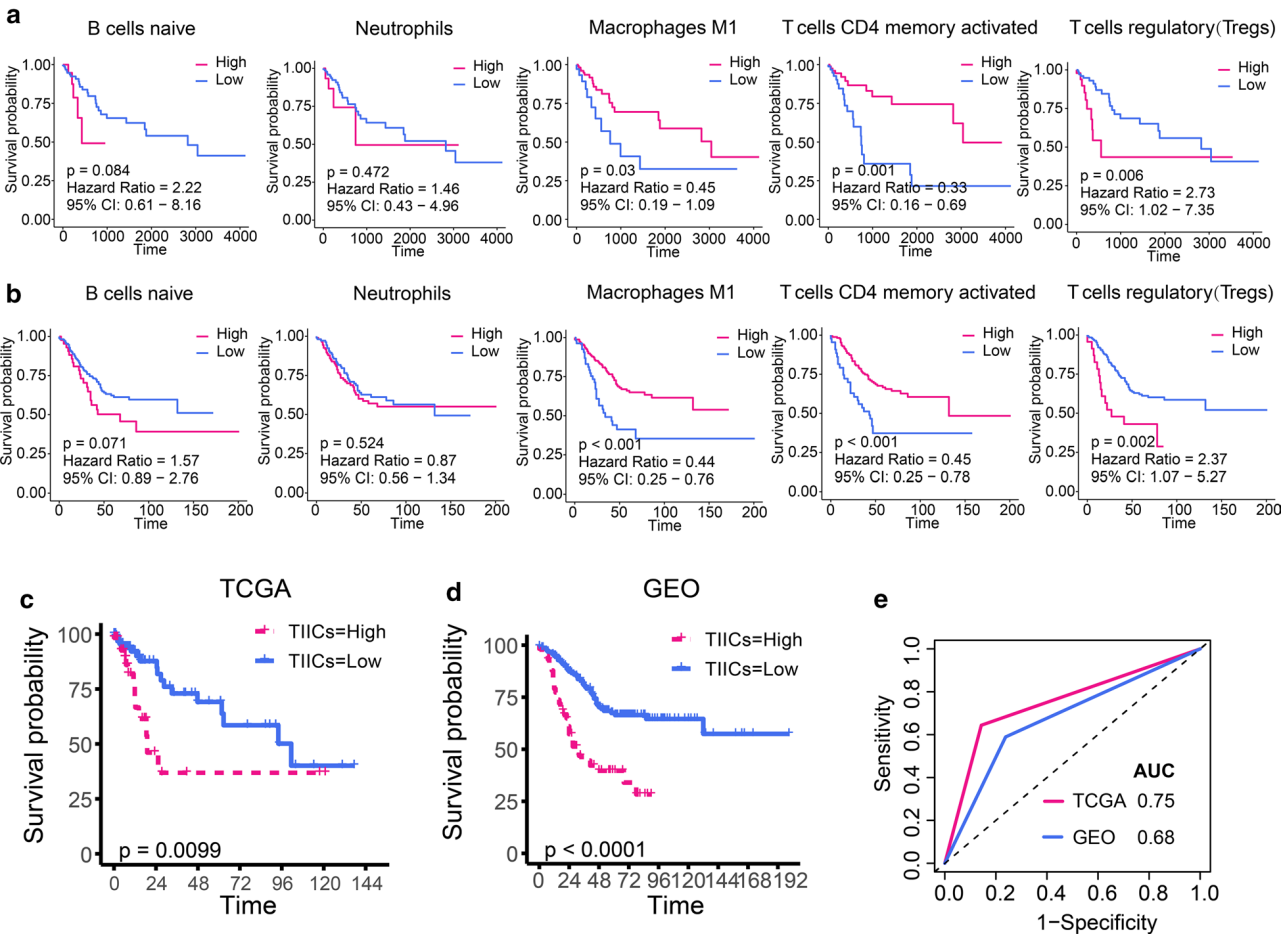
Development and validation of the tumor-infiltrating immune cell signature. **a** Univariate survival analysis identified survival-associated TIICs in patients with *KRAS* mutation based on training set. **b** Univariate survival analysis identified survival-associated TIICs in patients with *KRAS* mutation based on validation set. **c** Kaplan–Meier curves for patients with high- and low TIICs score in the training set. **d** Kaplan–Meier curves for patients with high- and low TIICs score in the validation set. **e** ROC curves for measuring the predictive value of the TIICs score in the training set and validation set

Given that TIICs were associated with *KRAS* mutations, we investigated whether TIICs were associated with survival in patients with *KRAS* mutations. We performed survival analysis to identify survival-associated TIICs in patients with *KRAS* mutation based on above differentially expressed TIICs. We observed that high abundance of macrophage M1 and activated CD4 memory T cell were associated with better prognosis while high abundance of Tregs was associated with poorer prognosis in patients with *KRAS* mutation (Fig. 3a). There was no significant correlation between the abundance of native B cells and neutrophils and prognosis (Fig. 3a). We validated these results using an independent validation set (GSE39582) which included the gene expression profile of 566 cases of CRC. Survival analysis likewise suggested that macrophage M1 and activated CD4 memory T cells were associated with better prognosis, whereas high abundance of Tregs was associated with poorer prognosis in patients with *KRAS* mutations (Fig. 3b). We further investigated the prognostic value of the above TIICs in *KRAS* wild-type patients. In the training set, only macrophage M1 was significantly associated with survival in *KRAS* wild-type patients (Additional file 5: Figure S5a, *p* = 0.008) and the prognostic value of macrophage M1 in *KRAS* wild-type patients could not validated by validation set (Additional file 5: Figure S5b, *p* = 0.185). These results suggested that macrophage M1, activated CD4 memory T cells and Tregs had a more robust prognostic value in the *KRAS*-mutant CRC. Next, we performed multivariate Cox regression analysis to identify independent prognostic TIICs and the results showed that macrophage M1, activated CD4 memory T cells and Tregs were independent prognostic TIICs for *KRAS*-mutant CRC (Additional file 9: Table S1). Therefore, collection of macrophage M1, activated CD4 memory T cells and Tregs were defined as the TIICs signature to predict prognosis of *KRAS*-mutant CRC. Based on the TIICs signature, we constructed a method to calculate scores of TIICs signature (TIICs score), which was calculated as follows: (-0.75 × abundance of macrophage M1) + (− 0.84 × abundance of activated CD4 memory T cells) + (0.93 × abundance of Tregs). The TIICs scores successfully distinguished *KRAS*-mutant CRC patients into high-risk or low-risk groups. Patients with high risk had significantly poorer overall survival compared with those with low risk in the TCGA dataset (Fig. 3c). The TIICs signature yielded similar results in *KRAS*-mutant CRC patients of validation set. Compared with those with low risk, high-risk patients had poorer OS (Fig. 3d). The AUC under ROC curve of TIICs scores for predicting OS in the training set and validation set were 0.75 and 0.68, respectively (Fig. 3e).

### Differentially expressed IRGs in *KRAS*-mutant and *KRAS* wile-type CRC patients

The expression of IRGs was compared to explore the immune molecular characteristics of CRC patients in the presence and absence of *KRAS* mutation. Among the 1951 IRGs, the edgeR algorithm identified 73 differentially expressed IRGs, of which 24 were up regulated and 49 down regulated in CRC with *KRAS* mutation (Additional file 6: Figure S6a). The pathways and biological processes influenced by differentially expressed IRGs were explored using enrichment analysis. The humoral immune response was the most significantly enriched pathway related to biological processes (Additional file 6: Figure S6b). Neuroactive ligand − receptor interaction, cytokine − cytokine receptor interaction, and Ras signaling pathway were the first three most significant pathways related to KEGG (Additional file 6: Figure S6c). Molecular functions enrichment analysis identified receptor ligand activity as the most frequent molecular function (Additional file 6: Figure S6d). Thus, these results indicate an association of a majority of differentially expressed IRGs with signal transduction. Results of protein–protein interaction (PPI) network analysis performed based on differentially expressed IRGs detected 133 edges and identified albumin (ALB), glucagon (GCG), leptin (LEP), insulin-like growth factor 2 (IGF2), CRP, and (pro-platelet basic protein) PPBP as core genes from the networks (Additional file 6: Figure S6e).

### Development and validation of the immune risk (Imm-R) model by intergrating TIICs and IRGs

**Fig. 4:**
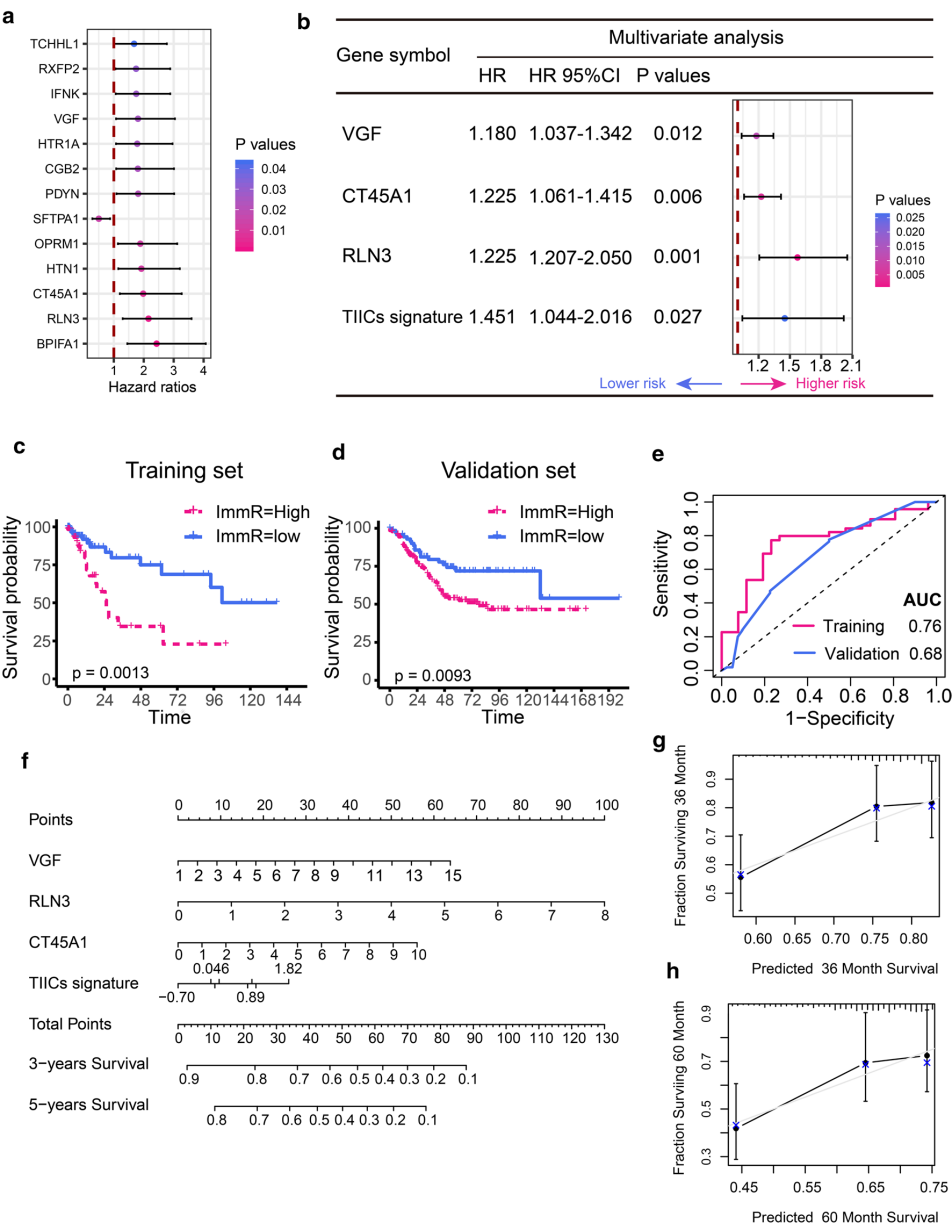
Development and validation of the immune risk (Imm-R) model.** a** Differentially expressed IRGs related with overall survival in univariate Cox regression analysis. *P* values are indicated by color scale on the side. Horizontal bars represent 95% confidence intervals. **b** Independent prognostic factors in multivariate COX regression analysis. *P* values are indicated by color scale on the side. Horizontal bars represent 95% confidence intervals. **c** Kaplan–Meier curves for patients with high- and low Imm-R score in the training set. **d** Kaplan–Meier curves for patients with high- and low Imm-R score in the validation set. **e** ROC curves for measuring the predictive value of the Imm-R model in the training set and validation set. **f** The nomogram of Imm-R model for predicting the three- and five-year survival probabilities. Points are assigned for four features. The score for each feature was calculated by drawing a line upward to the 'Points' line, and the sum of the four scores was 'Total Points'. The total points on the bottom scales correspond to the predicted three- and five-year survival. **g** The calibration plot of the nomogram predicting three-year survival. The x-axis is the nomogram-predicted survival and the y-axis is the actual survival. The reference line is 45° and indicates perfect calibration. **h** The calibration plot of the nomogram predicting five-year survival

Immune-related genes (IRGs) were reported to orchestrate tumor-associated immune responses. The integration of the TIICs signature and IRGs signature may enable more comprehensive assessment of immune status and more precise prognostic prediction. Preliminary screening of survival-associated IRGs using univariate Cox regression analysis revealed significant association of 13 out of 73 differentially expressed IRGs with OS (Fig. 4a). Importantly, majority of the survival-associated IRGs (12 out of 13) were identified as risk factors for poor prognosis. Further, independent prognostic factors were identified by multivariate COX regression analysis. Of the 13 genes subjected to multivariate COX regression model, three independent prognostic factors were identified, namely, VGF, relaxin 3 (RLN3), and cancer/testis antigen family 45 member A1 (CT45A1). The immune risk (Imm-R) model was constructed by intergrating TIICs signature and IRGs using multivariate COX regression. VGF, RLN3, CT45A1 and TIICs signature were all independent prognostic factors for *KRAS*-mutant CRC (all *p* < 0.05, Fig. 4b). In the Imm-R model, an immune risk score (Imm-R score) was generated using the formula: Imm-R score = (0.165 × VGF) + (0.453 × RLN3) + (0.203 × CT45A1) + (0.372 × TIICs signature). The Imm-R model could effectively distinguish *KRAS*-mutant CRC patients with discrete clinical outcomes (Fig. 4c). Patients at high-risk had significantly shorter survival compared to those at low-risk (*p* = 0.0013). We further validated the Imm-R model in *KRAS*-mutant CRC patients in validation set. The Imm-R model was powerful to distinguish *KRAS*-mutant CRC patients with good or bad prognosis. Patients with high-risk had significantly shorter OS compared with those with low risk in the validation set (*p* = 0.0096, Fig. 4d). The AUC under ROC curve of Imm-R model for predicting OS in the training set and validation set were 0.76 and 0.68, respectively (Fig. 4e). Nomogram is a user‑friendly graphical regression model with excellent applicability in clinical settings [24, 25]. To improve the usability of the Imm-R model, we constructed a nomogram to depict the Imm-R model better (Fig. 4f). The nomogram included above four features, and a point for each feature was assigned based on the scale on the top. The total score was defined as the sum of the points of the eight variables. By drawing a perpendicular line from the total point axis to the two-outcome axis, estimated three- and five-year OS probabilities could be obtained. To assess the goodness-of-fit of the nomogram, we compared the predicted three- and five-year survival probabilities to the actual three- and five-year survival probabilities using calibration plots (Fig. 4g, h). The calibration curve revealed good concordance between the predicted and observed probabilities in both of the three- and five-year survival probabilities. These results proved that the Imm-R model had very appropriate calibration.

### Association between immune infiltration and Imm-R model

**Fig. 5:**
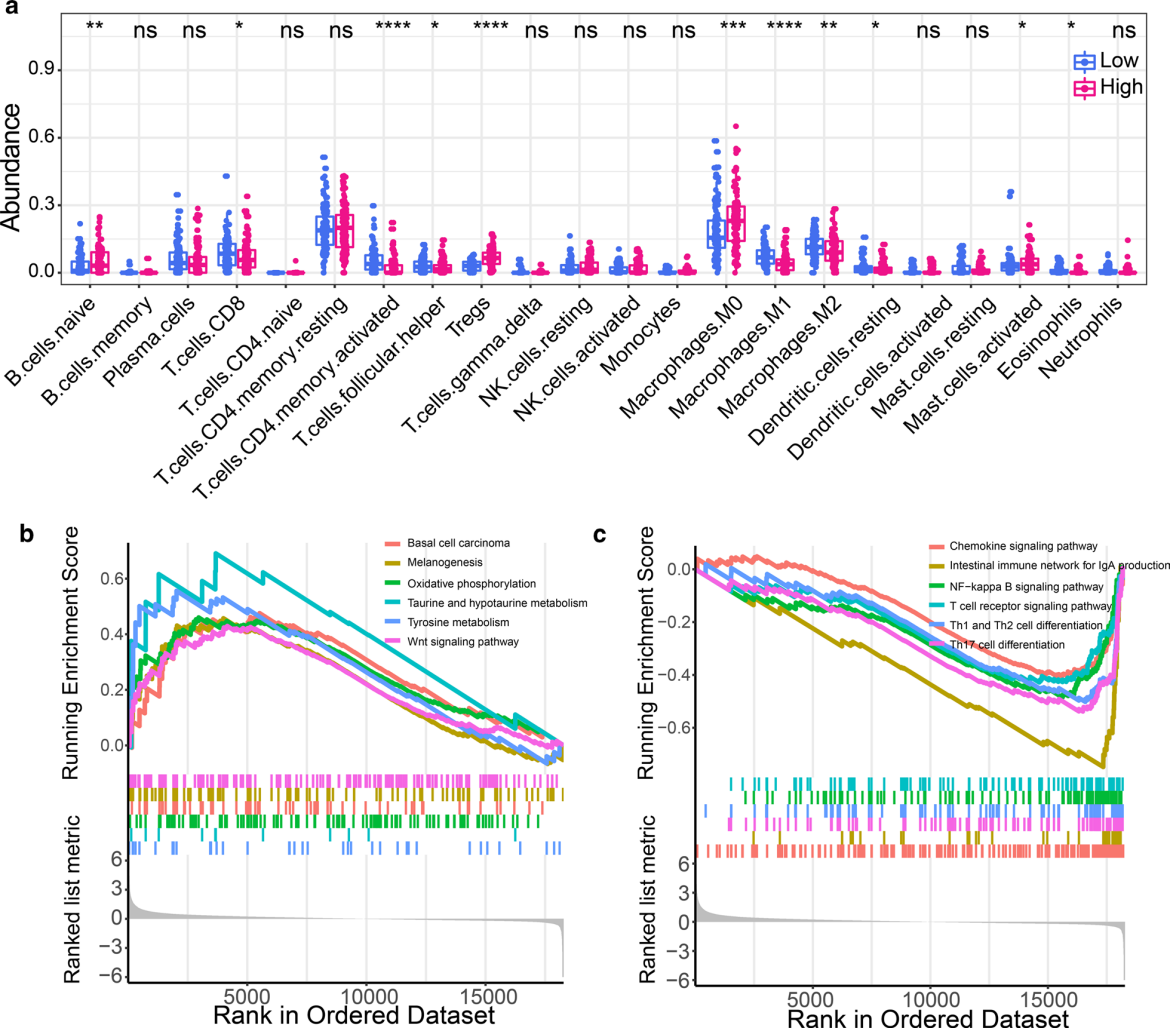
Association between immune infiltration and Imm-R model.** a** Abundance of immune cells in low- and high-risk CRC patients. **b** Pathways significantly enriched in CRC patients with high-risk. **c** Pathways significantly enriched in CRC patients with low-risk

The relationship between immune infiltration and Imm-R model was investigated by comparing the abundance of the 22 types of immune cells between the low- and high-risk CRC patients. The abundance of native B cells, Tregs, macrophage M0, activated mast cells were significantly increased in patients with high risk, while CD8 + T cells, activated CD4 memory T cell, follicular helper T cells, macrophage M1 and M2, resting dendritic cells and esoinophils were significantly decreased in patients with high risk (Fig. 5a). To explore the underlying biological mechanisms of the Imm-R model, we performed Gene Set Enrichment Analysis (GSEA). The results observed that several pathways related to cancer or metabolism were significantly activated in those high-risk patients, included Basal cell carcinoma, Wnt signaling pathway, melanogenesis and Taurine and hypotaurine metabolism, reflecting the active tumor metabolism in high-risk patients (Fig. 5b). Inversely, several pathways related to immune, such as chemokine signaling pathway, NF-kappa B signaling pathway and T cell recepter signaling pathway, were significantly down-regulated in high-risk patients, suggesting immunosuppression in high-risk patients (Fig. 5c). We further investigated the association between the expression of IRGs and TIICs. The IRGs significantly associated with macrophage M1 and activated CD4 memory T cell were summarized in Additional file 7: Figure S7. Interestingly, we found that FGF6 was significantly associated with the three TIICs mentioned above, which indicated that FGF6 might play an important role in the tumor microenvironment of CRC patients with KRAS mutation.

### Systemic immune and inflammatory state in CRC patients in the presence of *KRAS* mutation

The systemic impact of aberrant immune infiltration in localized tumor tissue was evaluated by comparing the level of immune cells, Igs, blood platelets, and acute phase proteins, such as CRP and hs-CRP in the blood of 335 CRC patients (101 patients tested positive for *KRAS*-mutation and 234 had *KRAS* wild-type). The clinical information related to the enrolled patients is listed in Table 1. The mean age of patients was 59 years. The proportion of patients with distant metastasis was significantly higher in *KRAS* wild-type patients compared to those harboring *KRAS* mutation (*p* = 0.02). Age, sex, tumor location, and TNM (T describes the size of the tumor and any spread of cancer into nearby tissue; N describes spread of cancer to nearby lymph nodes; and M describes metastasis) stage were similar between the two groups (*p* > 0.05). The systemic levels of hs-CRP, CRP, and IgM were significantly lower in *KRAS*-mutant compared to that of the *KRAS* wild-type patients (Additional file 8: Figure S8). The number of leukocyte, neutrophils, lymphocytes, blood platelets, lgM, lgA, C3, C4, helper T lymphocytes, inhibitory T lymphocytes, NK cells and B lymphocytes were similar between the *KRAS*-mutant and *KRAS* wild-type patients (*p* > 0.05).

**Table 1:** Characteristic of CRC patients with or without KRAS mutation

Characteristics	CRC patients		P-value
	KRAS-mutated (n=101)	KRAS wild-type (n=234)	
Age			0.61
[Median (IQR)] (year)	61 (49,68)	59.5 (51.25,67.75)	
Sex			0.85
Male	62 (30.5)	141 (69.5)	
Female	39 (29.5)	93 (70.5)	
Primary site			0.19
Rectum	49 (33.8)	96 (66.2)	
Left colon	22 (23.2)	73 (76.8)	
Transverse colon	11 (40.7)	16 (59.3)	
Right colon	19 (27.9)	49 (72.1)	
Pathological T classification			0.36
T1-2	15 (36.6)	26 (63.4)	
T3-4	86 (29.7)	204 (70.3)	
Pathological N classification			0.95
N0	53 (30.5)	121 (69.5)	
N1-2	44 (30.8)	99 (69.2)	
M classification			0.02*
M0	83 (43.5)	108 (56.5)	
M1	18 (27.3)	48 (72.7)	
Pathological stage			0.85
I-II	47 (30.5)	107 (69.5)	
III-IV	52 (31.5)	113 (68.5)	
Leukocyte (10^9^/L)	6.70±2.16	6.85±2.60	0.614
Blood platelet (10^9^/L)	283.94±99.25	292.55±108.71	0.495
Neutrophil (10^9^/L)	3.82±2.24	4.36±3.53	0.157
Lymphocyte (10^9^/L)	1.63±0.61	1.79±1.37	0.260
Albumin (g/L)	18.73±10.46	22.76±28.45	0.167
Total T lymphocyte (%)	65.77±9.76	65.58±1034	0.877
Helper T lymphocyte (%)	40.00±7.34	39.13±8.68	0.374
Suppressor t lymphocyte (%)	20.38±.82	20.67±7.27	0.739
Natural killer cell (%)	14.17±8.01	14.04±7.31	0.888
B-lymphocyte (%)	12.48±5.71	11.96±7.25	0.518
Immunoglobulin G (g/L)	11.86±3.12	11.54±2.98	0.369
Immunoglobulin M (g/L)	0.91±0.40	1.08±0.94	0.024*
Immunoglobulin A (g/L)	2.51±1.18	2.44±0.98	0.617
Complement C3 (g/L)	0.95±0.20	0.99±0.24	0.134
Complement C4 (g/L)	0.25±0.09	0.25±0.10	0.909
CRP (mg/L)	7.07±9.20	10.61±18.43	0.020*
hs-CRP (mg/L)	1.60±2.19	2.43±4.33	0.020*

*P<0.05 *CRP* C-reactive protein, *hs-CRP* high-sensitivity C-reactive protein

## Discussion

The benefits of immunotherapy have received immense research interest because of the impressive long-lasting response seen in several solid tumors [26]. In CRC, immune response and survival benefit are limited to mismatch-repair-deficient and microsatellite instability-high (dMMR–MSI-H) CRC patients, who account for only a small percentage of CRC patients. Thus, a deeper understanding of the immune landscape and identification of novel immunotherapeutic targets are needed. The present study systematically depicted the immune landscape and identified aberrant IRGs in *KRAS*-mutant and *KRAS* wild-type CRC patients. IRGs prognostic signature-based stratification effectively classified CRC patients into high- and low-risk groups with significantly evident differences in immune infiltration. This study provides a conceptual framework to understand the nature of immune infiltration in CRC in the context of *KRAS* mutation. This understanding might help interpret the probable responses to immunotherapy and treatment strategies designed to treat *KRAS*-mutant CRC patients.

*KRAS* mutation has been associated with immunosuppression in CRC. The presence of *RAS* mutation in CRC has been shown to down-regulate the IFNγ pathway, result in restricted CD8 + T cell activation [8, 27]. Immune checkpoint blockers, such as anti-PD-1 and anti-PD-L1 antibodies block the interaction between PD-1 and PD-L1, enhancing T cell activation that results in cytotoxic killing of tumor cells. However, inhibition of PD-L1 in *KRAS*-mutant CRC failed to bring about the desired result [28]. Several studies have explored the mechanism of immunosuppression and have provided insights to explain the mechanism of resistance to immunotherapy in *KRAS*-mutant CRC patients. Mutant *KRAS* inhibits the expression of interferon regulatory factor 2 (IRF2), a key transcription factor required for the activation of IFN-mediated responses [15]. Overexpression of IRF2 enhances sensitivity of *KRAS*-mutant CRC cells to anti-PD-1 therapy [15]. However, comprehensive analysis of aberrant IRGs and pathways associated with *KRAS* mutation in CRC still needs to be elucidated. Our study indicates down regulation of several immune and inflammatory pathways, such as NF-κB and T-cell receptor signaling pathways in *KRAS*-mutant CRC patients. The NF-κB signaling pathway is an important component of innate and adaptive immunity [29]. In innate immunity, upon activation of pattern recognition receptors (PRRs), NF-κB is crucial for the secretion of cytokines and for the production of perforin and IFN-γ in NK cells [30]. In adaptive immunity, the NF-κB signaling pathway is essential for the differentiation of B and T lymphocytes, and for the production of survival and maturation factors [31]. Inhibition of the NF-κB signaling pathway in the tumor microenvironment is a novel therapeutic target in immunotherapy. NF-κB-activating receptors are potential targets for combating the anti-inflammatory and regulatory effects of infiltrating regulatory T cells (Tregs) and functions as an important supportive therapy for checkpoint inhibitors [31]. In addition, metabolic pathways such as biosynthesis of amino acids and carbon metabolism were activated in *KRAS*-mutant CRC. Metabolic reprogramming, in which increased utilization of glucose and glutamine to support rapid growth is a hallmark of most cancers [32]. *KRAS*-driven metabolic rewiring occurs by up-regulating rate-limiting enzymes involved in amino acid, fatty acid, or nucleotide biosynthesis [33]. Targeting abnormal metabolic pathways may offer novel therapeutic strategies for the treatment of *KRAS* mutant CRC.

In CRC, *KRAS* mutation is associated with aberrant immune infiltration [34]. Our study found a significant decrease in the abundance of native B cells, neutrophils and macrophage M1, activated CD4 memory T cell in *KRAS*-mutant CRC. The the abundance of Tregs was significantly in *KRAS*-mutant CRC. A naive B cell is a B cell that has not been exposed to an antigen [35]. The antitumor activity of B cells is largely facilitated through IgG-mediated antigen presentation and activation of anti-tumor T cell responses [36]. In vivo, allogeneic IgG triggered a significantly more potent anti-tumor immune response than syngeneic IgG [37]. However, the functional role and mechanism of native B cells in tumor immunology remains unknown. Neutrophils are the first line of defense against pathogens. In the tumor microenvironment, tumor-associated neutrophils (TAN) exhibit a dual role in the form of N1 (tumor-suppressive) and N2 (tumor-promoting) phenotypes depending on the stage of disease progression [38]. Anti-tumor neutrophils activated by tumor cells bind to tumor cells, secrete cytotoxic mediators such as hydrogen peroxide (H_2_O_2_), and induce tumor cell apoptosis [39]. Interaction between neutrophils and T cells is essential to raise an appropriate anti-tumor immune response [40]. Neutrophils present antigens and provide accessory signals required for T cell activation [41]. Macrophages play a dual role in tumor immunity [42]. Of the two subtypes, M1 macrophages are differentiated from monocytes when exposed to Th1-type cytokines while M2 macrophages are differentiated under the influence of macrophage colony-stimulating factor (M-CSF), prostaglandin F (PGF) and vitamin D3 [43]. The M1 and M2 macrophages have distinct functions. M1 macrophages secrete higher levels of interleukin (IL)-12 and lower level of IL-10, and thereby contribute to the anti-tumor immune response. M2 macrophages produce immuno-suppressive cytokines such as IL-10, transforming growth factor-beta (TGF-β) and VEGF, resulting in the suppression of the immune surveillance system [42]. In the tumor microenvironment, T cells play a prominent role compared to B cells in cancer immunotherapy [36]. Activated CD4 memory T cell derived from CD4 memory T cells stimulated again by antigen [44]. Activated CD4 memory T cell undergo rapid expansion, eliciting a more effective and rapid immune response than the primary immune response [45]. The persistence of antitumor immunotherapy is related to the number of CD4 memory T cell [46]. Treg play a major role in orchestrating immunomodulation during CRC [47]. Treg cells can inhibit an anti-tumor specific immune response in patients with CRC and is associated with tumor progression during CRC [48]. Aberrant immune infiltration seen in *KRAS*-mutant CRC provides a promising ground for improving the response rate of immunotherapy. Further research is warranted to elucidate the interaction between immune and tumor cells to provide new targets for immunotherapy.

IRGs and TIICs can predict the prognosis of patients with CRC. Down-regulated M1 and up-regulated M2 macrophages are associated with poor prognosis in CRC [49]. A prognostic immunoscore model based on immune cells was established to predict OS in CRC patients [50]. However, the study was focused either on immune infiltration or on IRGs in CRC. The current study systematically analysed variation in immune infiltration and IRGs in *KRAS*-mutant CRC and established a prognostic model by intergrating TIICs and IRGs to determine the immune status of patients. The present model shows prognostic biomarkers that could be used to categorize patients to help improve the effectiveness of immunotherapy. The prognostic model consist of three IRGs, namely VGF, RLN3 and CT45A1. VGF is a neuroendocrine polypeptide secreted by neuroendocrine cells and functions to enhance neuronal growth and to prevent apoptosis [51]. VGF-expression influences the mechanism involved in counter regulating the decrease in functionality of T lymphocytes [52]. However, the functional role and mechanism of VGF in CRC remains unknown. RLN3 encodes relaxin-3, a peptide hormone belonging to the insulin superfamily [53]. RLN3 play an important role in the regulation of energy homeostasis and appetite [53]. Recent studies substantiate the role of RLN3 in development and tumorigenesis. Relaxins promote tumor growth and metastatic colonization in brain [54]. RLN3 is implicated in the prognosis of hepatocellular carcinoma (HCC) [55]. In phylogenetics, CT45A1 belongs to a new family of genes. CT45A1 and is aberrantly overexpressed in various types of cancer [56]. Overexpression of CT45A1 advances epithelial-mesenchymal transition, and enhances cell stemness, tumorigenesis, invasion, and metastasis. CT45-derived human leukocyte antigen (HLA) class I peptides efficiently activated patient-derived cytotoxic T cells and promoted tumor cell killing, indicating its potential as an immunotherapeutic target [57].

Cancer is a systematic disease in which the progression is driven not only by the underlying genetic alteration but also by complex systemic processes [58–60]. Interaction between the host and tumor plays an important role in cancer progression [61]. The cancerous state releases an abundance of proinflammatory cytokines into the circulation, resulting in systemic inflammation. Antigens are then expressed on the surface of tumor cells induce high levels of antibody and T cell response [62]. Aberrant inflammatory and immune responses are common in poorly differentiated and advanced CRC and are associated with a less favorable outcome [63]. Thus, immune recognition and inflammatory mechanism in cancer does not always result in protective immune response [64]. In the present study, relatively lower levels of systemic inflammation (low CRP and hs-CRP) and lgM were seen in *KRAS*-mutant compared to that of *KRAS* wild-type CRC patients. Of note, high serum CRP is associated with poor prognosis. Serum CRP levels also correlate with PD-L1 expression. Patients with lower serum CRP have a longer median time with regard to failure of immunotherapy compared to that of patients with higher levels of serum CRP [65]. IgM is released following initial contact with potential pathogens and is the first line of adaptive immune response [66]. Tumor-directed IgM antibodies directed against tumor-specific variants are promising agents for anti-tumor therapy [67]. In the present study, lower abundance of B cells in *KRAS*-mutant CRC patients corresponded with lower levels of serum IgM, indicating diminished immune infiltration related to inhibited antigen recognition and presentation. The use of serum CRP and IgM levels as predictable biomarker of immunotherapy in *KRAS*-mutant CRC patients remains to be elucidated.

Although the present findings provide new insights into the impact of *KRAS* mutation on the tumor microenvironment of CRC, there are limitations to our work. First, we identified three immune-related genes (VGF, RLN3 and CT45A1) which were differentially expressed and prognostically relevant in patients with *KRAS* mutations. But whether these three genes function exclusively through immune mechanisms in CRC remains undetermined. Second, the small sample size of the in-house RNA sequencing data used in this study may increase the probability of type II error. Third, the above findings may require further molecular biology experiments to confirm and clarify the underlying mechanisms.

## Conclusions

In summary, this study systematically revealed the immune landscape and profiled the IRGs in *KRAS*-mutant and *KRAS* wild-type CRC patients. Mutant *KRAS* in CRC was associated with suppressed immune pathways and immune infiltration. An Imm-R model by intergrating TIICs and IRGs was established to determine the immune status, and for prognostic prediction in *KRAS*-mutant CRC patients. This study thus provides a conceptual framework to understand the tumor immune microenvironments of CRC in the context of *KRAS* mutation and treatment strategies designed to treat *KRAS*-mutant CRC patients.

## Supplementary Information

**Additional file 1:** **Figure S1.** Somatic mutation landscape of colorectal cancer (CRC) patients.

**Additional file 2:** **Figure S2.** Gene set enrichment analysis for gene ontology terms.

**Additional file 3: Figure S3. **The abundance of 22 immune cells estimated by CIBERSORT between the *KRAS*-mutant and *KRAS* wild-type groups.

**Additional file 4:** **Figure S4. **Differential abundance of tumor-infiltrating immune cells between the *KRAS*-mutant and *KRAS* wild-type CRC.

**Additional file 5:** **Figure S5.** **a** Univariate survival analysis of TIICs in *KRAS* wild-type patients based on training set. **b** Univariate survival analysis of TIICs in *KRAS* wild-type patients based on validation set.

**Additional file 6:** **Figure S6. **Differentially expressed immune-related genes (IRGs) in CRC in the presence and absence of *KRAS* mutation.

**Additional file 7:** **Figure S7. **The association between the expression of IRGs and TIICs.

**Additional file 8: Figure S8. **Systemic immune and inflammatory state in colorectal cancer (CRC) with Kirsten rat sarcoma viral oncogene homolog (*KRAS*) mutation.

**Additional file 9: Table S1.** Multivariate analyses of prognostic tumor-infiltrating immune cells in patients with KRAS-mutation

## Data Availability

The datasets analysed during the current study are available from the corresponding author on reasonable request. All data analysed during this study are included in this published article (Additional files:1:8).

## Abbreviations

CRC: Colorectal cancer
IRGs: Immune-related genes
hs-CRP: Hypersensitive C-reactive protein
KRAS: Kirsten rat sarcoma viral oncogene homolog
MAPK: Mitogen-activated protein kinase
VEGF: Vascular endothelial growth factor
RTKs: Receptor tyrosine kinases
GTP: Guanosine triphosphate
GDP: Guanosine diphosphatase
EGFR: Epidermal growth factor receptor
CIRC: Co-ordinate Immune Response Cluster
MDSCs: Myeloid-derived suppressor cells
TCGA: The Cancer Genome Atlas
MAF: Mutation Annotation Format
GEO: Gene Expression Omnibus
CRP: C-reactive protein
NK: Natural killer
TIMER: Tumor immune estimation resource
TMM: Trimmed mean of M-values
FDR: False discovery rate
GSEA: Gene set enrichment analysis
KEGG: Kyoto Encyclopedia of Genes and Genomes
GO: Gene ontology
OS: Overall survival
PI: Prognosis index
APC: Adenomatous polyposis coli
TP53: Tumor protein 53
NF-κB: Nuclear factor kappa-B
PPI: Protein–protein interaction
ALB: Albumin
GCG: Glucagon
LEP: Leptin
IGF2: Insulin-like growth factor 2
PPBP: Pro-platelet basic protein
RLN3: Relaxin 3
CT45A1: Cancer/testis antigen family 45 member A1
TP63: Tumor protein P63
TNM: T describes the size of the tumor and any spread of cancer into nearby tissue
N: Describes spread of cancer to nearby lymph nodes
M: Describes metastasi
dMMR–MSI-H: Mismatch-repair-deficient and microsatellite instability-high
IRF2: Interferon regulatory factor 2
PRRs: Pattern recognition receptors
Tregs: Regulatory T cells
TAN: Tumor-associated neutrophils
H2O2: Hydrogen peroxide
M-CSF: Macrophage colony-stimulating factor
PGF: Prostaglandin F
IL: Interleukin
TGF-β: Transforming growth factor-beta
HCC: Hepatocellular carcinoma
HLA: Human leukocyte antigen
