# Supplementary figures and images for: Immune landscape and prognostic immune-related genes in KRAS-mutant colorectal cancer patients
Source: J Transl Med. 2021 Jan 7;19:27. doi: 10.1186/s12967-020-02638-9 (PMC7789428; doi:10.1186/s12967-020-02638-9)

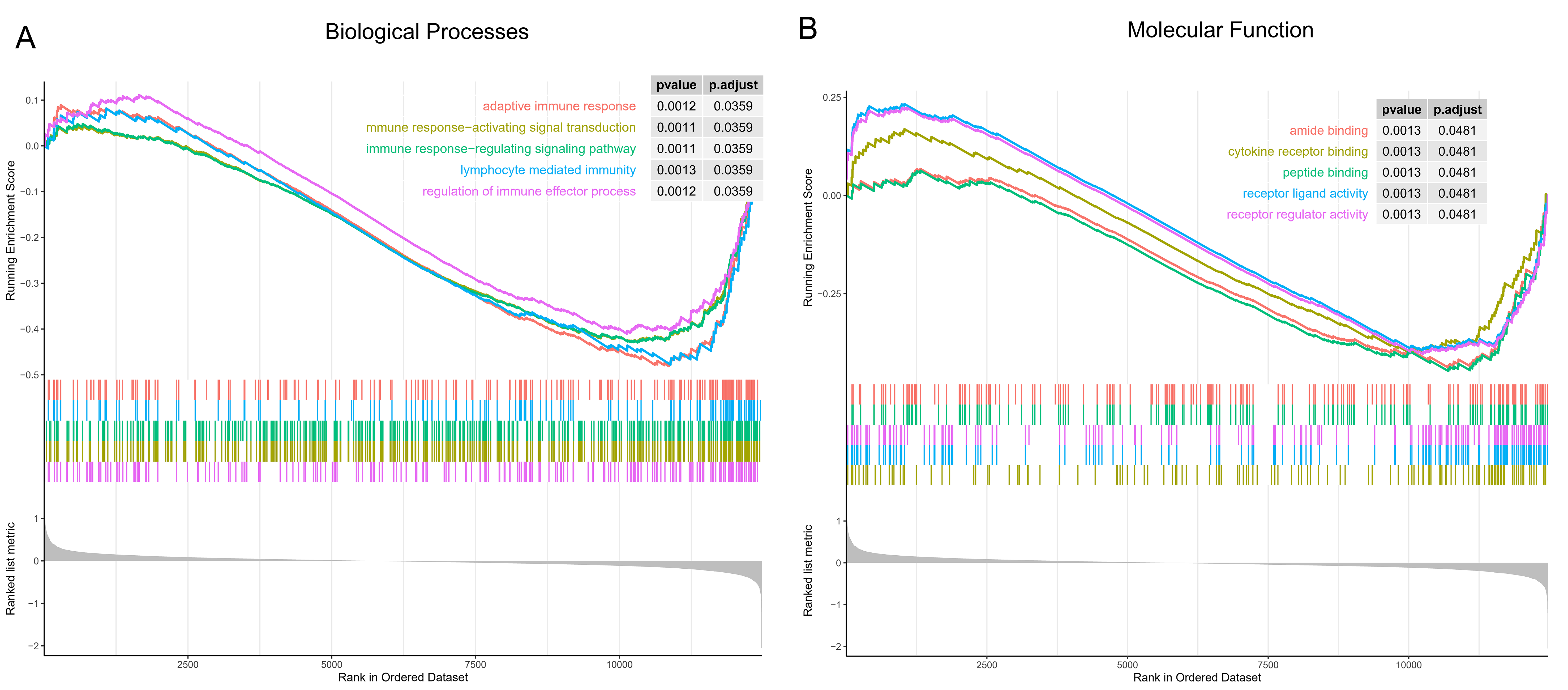

Supplement: Supplementary file 2 — Additional file 2: Figure S2. Gene set enrichment analysis for gene ontology terms. [file 12967_2020_2638_MOESM2_ESM.tif]

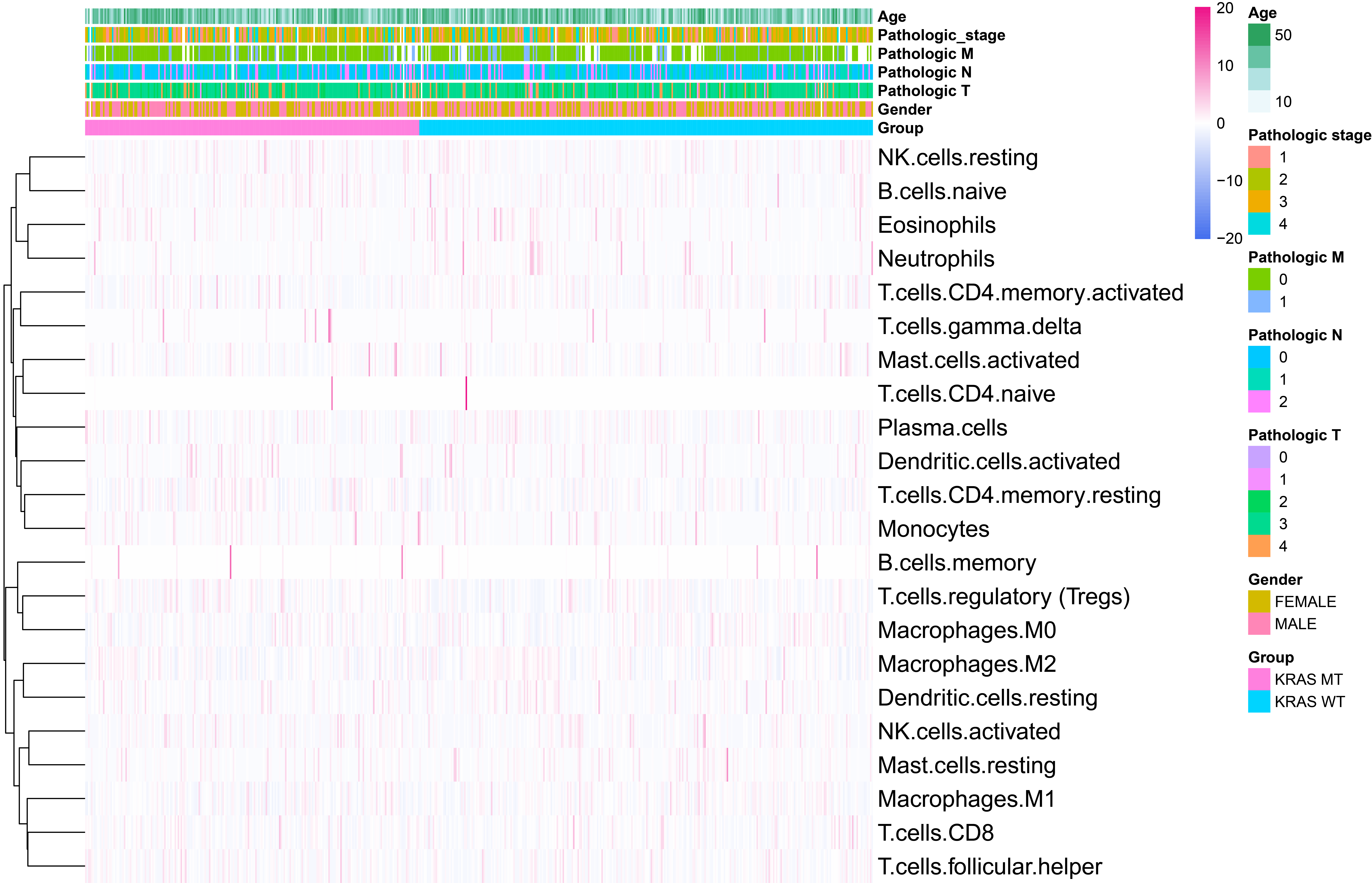

Supplement: Supplementary file 3 — Additional file 3: Figure S3. The abundance of 22 immune cells estimated by CIBERSORT between the KRAS-mutant and KRAS wild-type groups. [file 12967_2020_2638_MOESM3_ESM.tif]

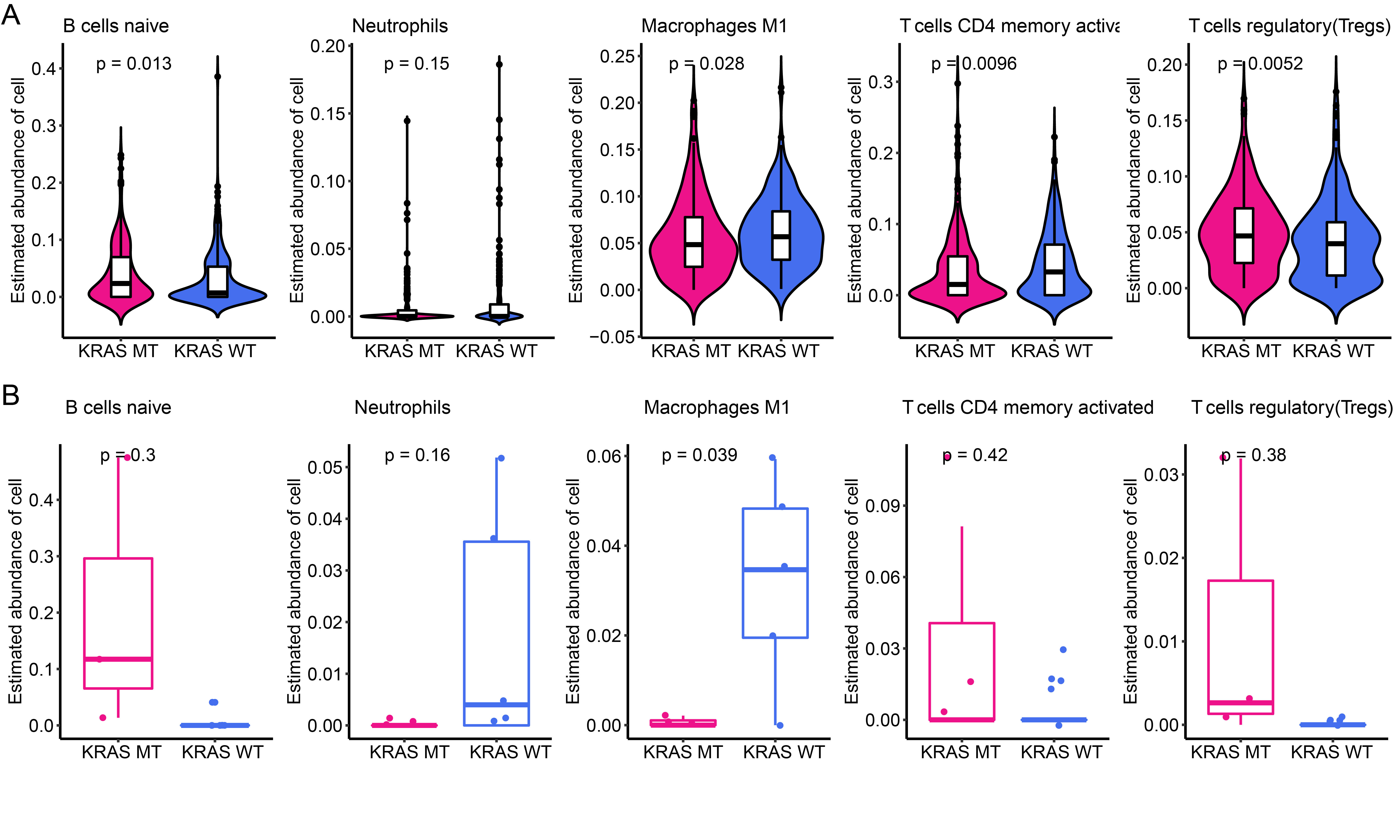

Supplement: Supplementary file 4 — Additional file 4: Figure S4. Differential abundance of tumor-infiltrating immune cells between the KRAS-mutant and KRAS wild-type CRC. [file 12967_2020_2638_MOESM4_ESM.tif]

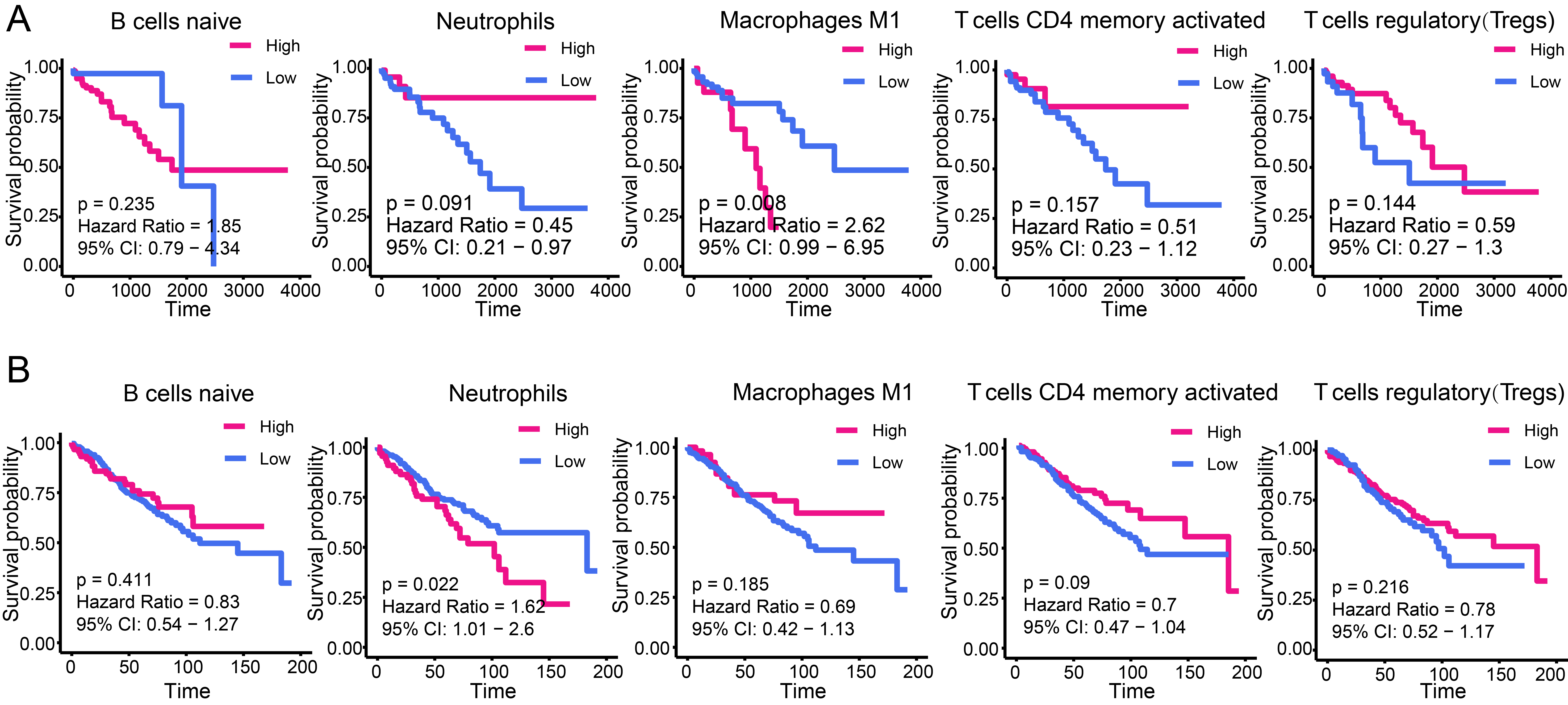

Supplement: Supplementary file 5 — Additional file 5: Figure S5. a Univariate survival analysis of TIICs in KRAS wild-type patients based on training set. b Univariate survival analysis of TIICs in KRAS wild-type patients based on validation set. [file 12967_2020_2638_MOESM5_ESM.tif]

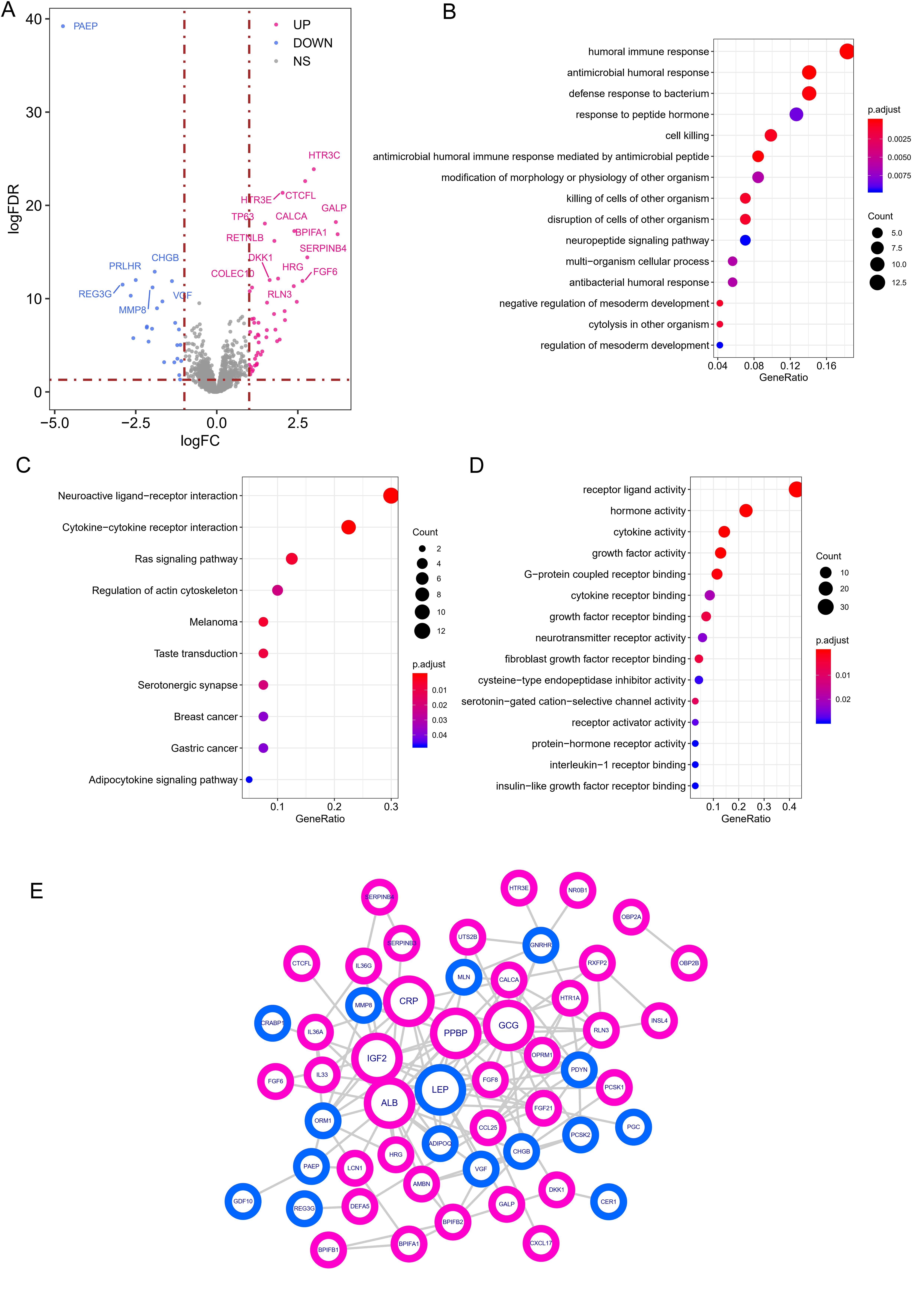

Supplement: Supplementary file 6 — Additional file 6: Figure S6. Differentially expressed immune-related genes (IRGs) in CRC in the presence and absence of KRAS mutation. [file 12967_2020_2638_MOESM6_ESM.tif]

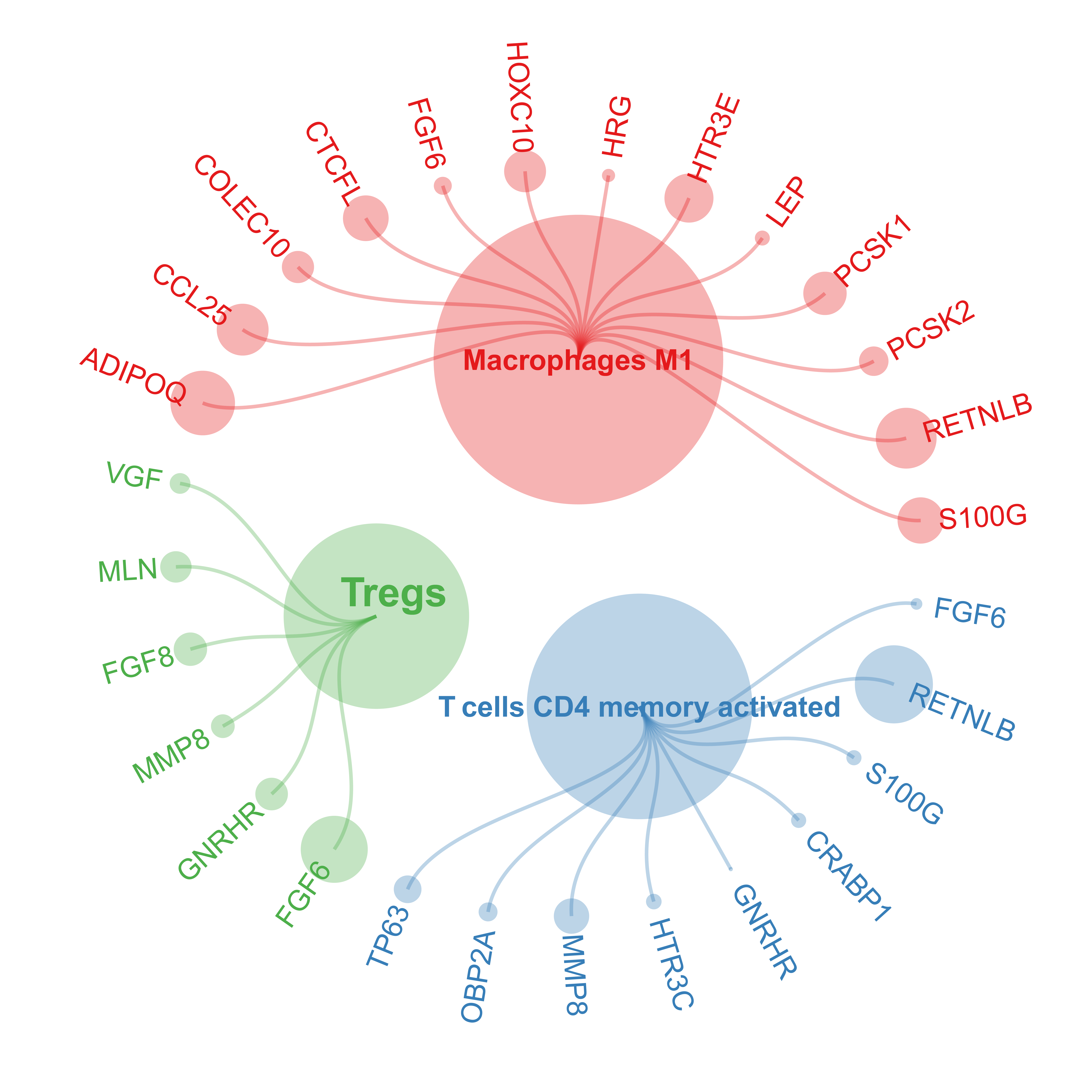

Supplement: Supplementary file 7 — Additional file 7: Figure S7. The association between the expression of IRGs and TIICs. [file 12967_2020_2638_MOESM7_ESM.tif]

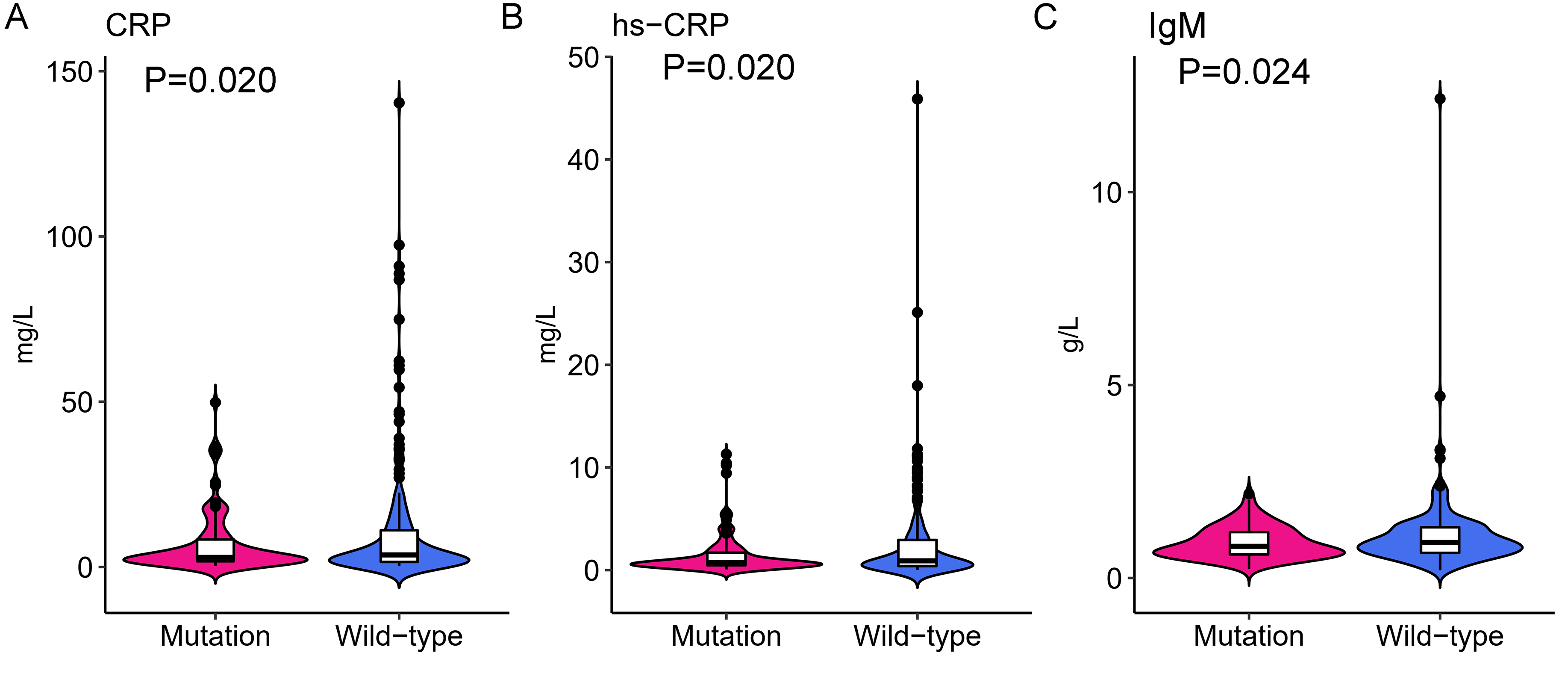

Supplement: Supplementary file 8 — Additional file 8: Figure S8. Systemic immune and inflammatory state in colorectal cancer (CRC) with Kirsten rat sarcoma viral oncogene homolog (KRAS) mutation. [file 12967_2020_2638_MOESM8_ESM.tif]
