# Supplementary material for: Immune landscape and prognostic immune-related genes in KRAS-mutant colorectal cancer patients
Source: J Transl Med. 2021 Jan 7;19:27. doi: 10.1186/s12967-020-02638-9 (PMC7789428; doi:10.1186/s12967-020-02638-9)
Supplement: Supplementary file 9 — Additional file 9: Table S1. Multivariate analyses of prognostic tumor-infiltrating immune cells in patients with KRAS-mutation [file 12967_2020_2638_MOESM9_ESM.docx]

Table S1: Multivariate analyses of prognostic tumor-infiltrating immune cells in patients with KRAS-mutation

| Variables | Multivariate COX analysis | | |
| --- | --- | --- | --- |
|  | HR | 95%CI | P-values |
| T cells CD4 memory activated | 0.43 | 0.20-0.93 | 0.0319 |
| Tregs | 2.55 | 1.10-5.95 | 0.0300 |
| Macrophages M1 | 0.47 | 0.22-0.99 | 0.0485 |
